# Defining a mood stabiliser: novel framework for research and clinical practice

**DOI:** 10.1192/bjo.2018.36

**Published:** 2018-07-20

**Authors:** Gin S. Malhi, Richard Porter, Lauren Irwin, Amber Hamilton, Grace Morris, Darryl Bassett, Bernhard T. Baune, Philip Boyce, Malcolm J. Hopwood, Roger Mulder, Gordon Parker, Zola Mannie, Tim Outhred, Pritha Das, Ajeet B. Singh

**Affiliations:** Treatment Algorithm Group (TAG), Academic Department of Psychiatry, Northern Sydney Local Health District, Sydney Medical School Northern, University of Sydney, Australia and CADE Clinic, Royal North Shore Hospital, Northern Sydney Local Health District, Australia; Treatment Algorithm Group (TAG) and Department of Psychological Medicine, University of Otago – Christchurch, New Zealand; Treatment Algorithm Group (TAG), Academic Department of Psychiatry, Northern Sydney Local Health District, Sydney Medical School Northern, University of Sydney, Australia and CADE Clinic, Royal North Shore Hospital, Northern Sydney Local Health District, Australia; Treatment Algorithm Group (TAG), Academic Department of Psychiatry, Northern Sydney Local Health District, Sydney Medical School Northern, University of Sydney, Australia and CADE Clinic, Royal North Shore Hospital, Northern Sydney Local Health District, Australia; Treatment Algorithm Group (TAG), Academic Department of Psychiatry, Northern Sydney Local Health District, Sydney Medical School Northern, University of Sydney, Australia and CADE Clinic, Royal North Shore Hospital, Northern Sydney Local Health District, Australia; Treatment Algorithm Group (TAG) and University of Western Australian Medical School, Australia, Faculty of Health and Medical Science, University of Western Australia, Australia; Treatment Algorithm Group (TAG) and Discipline of Psychiatry, University of Adelaide, Australia; Treatment Algorithm Group (TAG) and Discipline of Psychiatry, Sydney Medical School, Australia and Westmead Clinical School, University of Sydney, Australia; Treatment Algorithm Group (TAG) and Department of Psychiatry, University of Melbourne, Australia; Treatment Algorithm Group (TAG) and Department of Psychological Medicine, University of Otago – Christchurch, New Zealand; Treatment Algorithm Group (TAG) and School of Psychiatry, University of New South Wales, Australia and Black Dog Institute, Australia; Treatment Algorithm Group (TAG), Academic Department of Psychiatry, Northern Sydney Local Health District, Sydney Medical School Northern, University of Sydney, Australia and CADE Clinic, Royal North Shore Hospital, Northern Sydney Local Health District, Australia; Treatment Algorithm Group (TAG), Academic Department of Psychiatry, Northern Sydney Local Health District, Sydney Medical School Northern, University of Sydney, Australia and CADE Clinic, Royal North Shore Hospital, Northern Sydney Local Health District, Australia; Treatment Algorithm Group (TAG), Academic Department of Psychiatry, Northern Sydney Local Health District, Sydney Medical School Northern, University of Sydney, Australia and CADE Clinic, Royal North Shore Hospital, Northern Sydney Local Health District, Australia; Treatment Algorithm Group (TAG) and School of Medicine, IMPACT Strategic Research Centre, Deakin University, Barwon Health, Australia

**Keywords:** Bipolar disorder, mood disorders, mood stabilisers, psychopharmacology, prophylaxis

## Abstract

The term ‘mood stabiliser’ is ill-defined and lacks clinical utility. We propose a framework to evaluate medications and effectively communicate their mood stabilising properties – their acute and prophylactic efficacy across the domains of mania and depression. The standardised framework provides a common definition to facilitate research and clinical practice.

**Declaration of interest:**

The Treatment Algorithm Group (TAG) was supported logistically by Servier who provided financial assistance with travel and accommodation for those TAG members travelling interstate or overseas to attend the meeting in Sydney (held on 18 November 2017). None of the committee were paid to participate in this project and Servier have not had any input into the content, format or outputs from this project.

The term ‘mood stabiliser’ has become ubiquitous when discussing the management of bipolar disorders but remarkably, despite its widespread use, there are no standard parameters by which to define the term. This is why not a single medication has an official indication as a ‘mood stabiliser’ *per se*. The term was originally used to refer to pharmacotherapies, such as lithium, that prevented the recurrence of mood episodes, but over time it has evolved to encompass drugs that decrease the frequency and severity of manic or depressive episodes, and now even includes medications that only have efficacy against mood disorder symptoms.^1^^,^^2^ Thus, presently, mood stabiliser is a loosely applied term that can be used to describe almost any of the medications prescribed for the treatment of bipolar disorder,^3^ and there is no clear consensus as to what constitutes a mood stabiliser, and what properties are essential.

The absence of a standard definition has led to medications that lack mood stabilising properties being given the moniker. For example, the antipsychotic olanzapine is one of the three most commonly used maintenance medications for the treatment of bipolar disorder (along with lithium and valproate) and has regulatory approval for long-term maintenance treatment of bipolar disorder, but there is little evidence to support its prescription for long-term prophylaxis.^4^ In addition, prescribing olanzapine for extended periods of time is now known to be associated with significant risks, such as metabolic syndrome.^5^ This highlights another corollary of the broad and non-specific adoption of the term mood stabiliser, in that it allows the prescription of agents far beyond their initial indications and putative usefulness.

At present, many medications labelled as mood stabilisers by commercial entities, and regarded as such by clinicians, have only shown short-term efficacy at best (against either manic or depressive symptoms). In contrast, their use has expanded to include chronic and indefinite administration – without substantive evidence of long-term effectiveness. Medications demonstrate differential actions when administered acutely for short periods of time, as compared with chronic use over the course of an illness, because different mechanisms are in play. It is also highly likely that a separate set of processes underpin recovery from an acute episode as compared with the prevention of future episodes. Thus, evidence of acute efficacy does not necessarily inform long-term efficacy and probably has little bearing on long-term prophylaxis, and, therefore, any such extrapolation from one phase of treatment to another is likely to be erroneous.

Clinically, the non-descript application of the term mood stabiliser has rendered it meaningless with respect to connoting pharmaceutical effect. Lithium, for example, the gold-standard of mood stabilisers because of its ability to ameliorate both acute mania and, in the long term, prevent the reoccurrence of mood episodes, possesses a starkly different therapeutic profile to other medications that also enjoy aggrandisement as ‘mood stabilisers’. For instance, lamotrigine, which has no effect on mania; aripiprazole, which can exacerbate acute depression; risperidone, which can successfully counter acute mania but have little evidence to suggest prophylactic efficacy; and ziprasidone, which similarly has scarce evidence to support long-term use and may also exacerbate acute depression, are all, at times, described as ‘mood stabilisers’. Consequently, there is considerable debate concerning the ongoing, indiscriminate use of the term, with some suggesting that it be abandoned altogether.^6^ However, its widespread familiarity and established use does not allow for such a tidy solution. Indeed, the descriptor is now ingrained in the lexicon of bipolar disorder management strategies, and both patients and clinicians are loathed to discontinue its use. Perhaps one of the most important reasons is that patients find the description of any treatment as a ‘mood stabiliser’ comforting and hopeful. These are immensely important qualities and, as recent research has shown, instilling hope improves outcome and the effectiveness of therapies. It is therefore necessary to recapture the term and devise a standard definition for what an ideal mood stabiliser should achieve in the management of bipolar disorder and what the minimum requirements should be for a medication to earn this recognition.

## Method

In order to define the term mood stabiliser, a committee comprising clinical and academic experts from Australia and New Zealand was convened. The committee, called the Treatment Algorithm Group (TAG), met in Sydney in November 2017. The committee was chaired by G.S.M. and supported by a multidisciplinary research team (G.M., A.H., L.I., Z.M., P.D., T.O.) independent of any professional bodies or pharmaceutical companies. It was facilitated academically by the University of Sydney and logistically by an unrestricted educational grant from Servier.

The committee first determined whether to retain the term mood stabiliser. This was agreed unanimously. Therefore, the committee then proceeded to examine how to best define the term so that it meaningfully informs clinical practice and future research.

## Results

### Defining a mood stabiliser

A framework was proposed that aimed to ensure accurate clinical utility of the term mood stabiliser and encourage research that better defines the actions of agents on pathological fluctuations of mood. In the research domain, a standardised template with which to evaluate pharmacological treatments should facilitate the development and assessment of interventions for bipolar disorder, and provide meaningful long-term mood stability to individuals with the illness.

### The ideal

**Fig 1.**
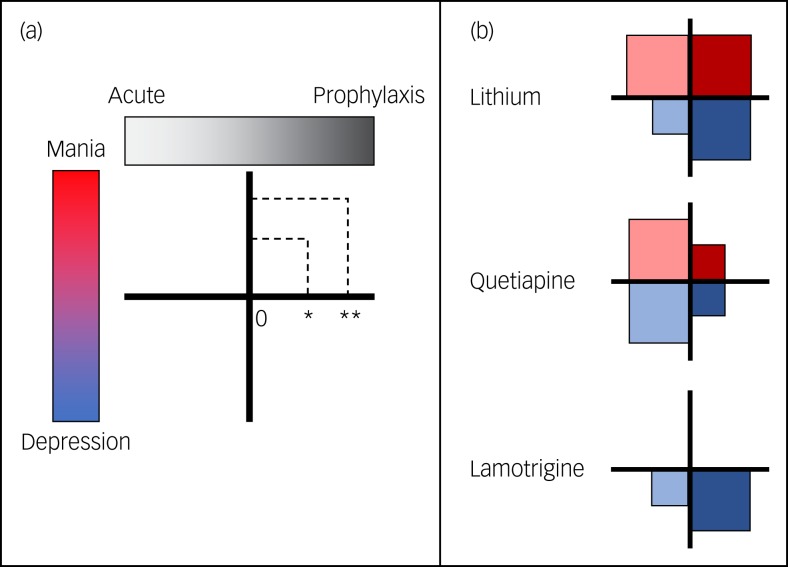
(a) The proposed framework appraises the efficacy of medications across four domains: their acute and prophylactic effectiveness against mania and depression. The size of the square in each domain reflects the efficacy of the medication. No square would indicate no effect (0), a small square indicates modest efficacy (*) and a large square indicates significant efficacy (**; as good as it gets). (b) Examples of the framework in action. At a glance, the framework communicates that lithium has significant efficacy in three domains (i.e. acute mania, prophylaxis against mania and prophylaxis against depression). Its efficacy in treating acute depression is rated as modest – partly because it takes considerable time to take effect. In contrast, quetiapine is more effective in the short term, but has only modest long-term efficacy, while lamotrigine is only effective in the treatment of bipolar depression, and more so for prophylaxis.

An ideal mood stabiliser should possess long-term prophylactic efficacy against both mania and depression, with minimal side-effects. The real world, however, is rarely ideal. Indeed, few medications would meet this ideal at present – with lithium clearly leading the way. Therefore, this paper presents an operational definition of mood stabilisers. In order to (a) capture more accurately the ‘mood stabilising profile’ of extant medications, (b) provide a framework against which the effects of future medications can be gauged and grouped on the basis of their mood stabilising properties, and (c) ensure that this information is communicated easily and effectively among clinicians and researchers, a framework has been proposed that evaluates the properties of a medication over four intersecting domains that capture the acute and prophylactic effects on depressive and manic symptoms (Fig. 1).

### Four domains

Collectively, the acute and prophylactic perspective on manic and depressive symptoms creates four ‘domains’ within which symptoms have an impact on individuals with bipolar disorder. Of course, in reality, there are also mixed presentations (which are as yet poorly defined and understood) and significant comorbidities in the form of anxiety symptoms, all of which interplay with personality. But for the purposes of defining the pharmacological actions of mood stabilising agents, the proposed framework focuses only on the relatively pure forms of mood presentations (depression and mania).

Within each domain, a medication is graded according to its potency – none (0), partial (*) or substantial (**). Acute and prophylactic efficacy has been illustrated as a spectrum because in reality it is difficult to identify distinct phases in the longitudinal course of an illness. Typically, the acute period is defined as the time until recovery, which is customarily formalised as 2 months after symptoms have remitted.^7^ Prophylaxis is the long-term maintenance that begins once a patient has recovered. For chronic illnesses, prophylactic treatments are regularly prescribed for an individual's entire life. Practically, and for the purposes of this framework, acute treatments are those taken to get well, whereas prophylactic treatments maintain wellness. Therefore, efficacious treatments reduce the frequency and severity of episodes and increase the duration of euthymia.

### Recommendation

Fundamentally, there are two aspects that must be considered when evaluating the suitability of a medication as a mood stabiliser for the treatment of bipolar disorder – mania and prophylaxis.

#### Mania

Mania defines bipolar disorder and distinguishes it from depressive disorders. Therefore, it is mandatory for a mood stabiliser to have efficacy against manic symptoms. That is, a medication that only displays efficacy within depressive domains and confers no benefit whatsoever against manic symptoms cannot be regarded as a mood stabiliser for the purposes of managing bipolar disorder. It may be regarded more broadly as having mood stabilising properties, for instance in the context of major depressive disorders, but in such cases it is essentially acting as an antidepressant – albeit one that may have some additional prophylactic properties with respect to depression. However, given that depression is typically more common throughout the course of bipolar disorder, a bipolar mood stabilising agent should also possess some degree of efficacy against depression to be a clinically useful mood stabiliser.

#### Prophylaxis

The chronic nature of bipolar disorder that confers life-long vulnerability for the development of mood episodes means that, although the focus of many medications and treatment guidelines is the amelioration of acute symptoms, it is the maintenance of euthymia and effective prophylaxis that ultimately ensures optimal outcomes. Hence, it is the successful treatment of this component of the illness that is of greatest importance to individuals with the disorder and, as such, mood stabilisers should be mandated to demonstrate prophylactic efficacy in maintaining mood stability and preventing relapses. Medications that are only effective in the acute phase cannot be regarded as genuine mood stabilisers. That is, although some agents may restore mood stability acutely (remission of manic or depressive symptoms), this should not qualify them as mood stabilisers.

### Additional perspectives

The proposed framework for gauging the mood stabilising properties of medications is also useful because it highlights aspects of mood stability and the additive effects of medications that also require further consideration and examination. For example, the nature of euthymia needs to be more rigorously defined. This is because many patients commonly complain that even when well (euthymic) they feel blunted to all emotions because of the effect of ongoing treatment. This ‘side-effect’, alongside many others, naturally has an impact on the individual's quality of life and deters from adherence to treatment. Furthermore, in addition to emotion, there are other domains that are affected by mood disorders, namely cognition and acitivity,^8^ which also need to be incorporated into future iterations of this model (detailed discussion of these is beyond the scope of this article). Thus, subjective quality of life, tolerability, and effects on cognition and activity also need to be considered when determining whether a medication is a potential mood stabiliser. These considerations apply to the effectiveness of medications rather than their efficacy, and it is the latter that should first determine whether an agent is suitable to be described as a mood stabiliser.

Another important consideration is that medications that create mixed mood states are clearly unsuitable for use as mood stabilisers.^9^ Interestingly, antidepressants can sometimes cause mixed states and, along a similar line of thinking, drugs that exacerbate any domain should not be regarded as mood stabilisers. Specifically, medications that ameliorate depression at the expense of exacerbating mania do not stabilise mood. However, drugs that are effective in some domains may perhaps be suitable as adjunctive therapies when trying to achieve mood stabilisation in the first place.

## Discussion

The benefit of the proposed framework is that it provides a clear picture of an agent's efficacy within the context of mood stabilisation. Ready adoption of the framework will allow for straightforward decision-making in both clinical and research settings. Indeed, for clinicians, the clear communication of a medication's efficacy across the domains of the framework has the potential to shift current prescribing practice towards medications that improve a patient's outcomes in the long-term. For instance, it is now widely known that many of the agents that are successful in treating acute mania are not suited to long-term use because of metabolic side-effects. But, in practice, there is still considerable inertia and reluctance to modify and tailor treatment. The framework could provide renewed impetus for such thinking and action, as it will provide guidance for clinicians to refine their use. It is important to note, however, that this framework is working against the natural order, in that it is taking an established term and altering its meaning. As such, while it would be beneficial for guidelines and researchers to adopt the framework, this will take a concerted effort. Ultimately, the framework has been designed for simple and effective communication, so that it can be easily included on a label or in a product's information statement, such that industry and government may adopt its usage in future.

It is necessary to consider the strength of available evidence when evaluating potential mood stabilisers, and here the proposed framework is a starting point that offers a template for future research. Many medications currently used in the maintenance treatment of bipolar disorder have not been adequately assessed over longer periods of time (5–10 years). Given the requirement that mood stabilisers provide life-long relief from mood fluctuations, there should be more robust and real-world evidence that a drug has a substantial effect before it is graded as such. Research assessing the prophylactic efficacy of medications for mood stability should ideally impose a minimum of 3 years’ evaluation, given that the natural history for bipolar disorder anticipates recurrence after 1.5–3 years. It is imperative that more research of this nature emerges to reliably classify pharmacotherapies as mood stabilisers.
